# Principles of genome activation in the early embryo

**DOI:** 10.1016/j.gde.2023.102062

**Published:** 2023-06-18

**Authors:** Coral Y Zhou, Rebecca Heald

**Affiliations:** Department of Molecular and Cell Biology, University of California, Berkeley, CA 94720, USA

## Abstract

A major hurdle in an embryo’s life is the initiation of its own transcriptional program, a process termed Zygotic Genome Activation (ZGA). In many species, ZGA is intricately timed, with bulk transcription initiating at the end of a series of reductive cell divisions when cell cycle duration increases. At the same time, major changes in genome architecture give rise to chromatin states that are permissive to RNA polymerase II activity. Yet, we still do not understand the series of events that trigger gene expression at the right time and in the correct sequence. Here we discuss new discoveries that deepen our understanding of how zygotic genes are primed for transcription, and how these events are regulated by the cell cycle and nuclear import. Finally, we speculate on the evolutionary basis of ZGA timing as an exciting future direction for the field.

## Introduction

For sexually reproducing organisms, embryogenesis begins following the fertilization of an egg by a sperm. However, the embryo does not acquire its own identity until it starts transcribing its own genome in a process called Zygotic Genome Activation (ZGA) [1,2]. ZGA begins as a specialized set of transcription factors called pioneer factors bind to nucleosomes at promoters and enhancers, allowing downstream transcription activators to access the DNA and leading to an increase in RNA polymerase II (Pol II) activity [3]. The misregulation of ZGA timing or activity leads to developmental delay, arrest, or even embryo death [1].

Classic studies in *Xenopus* demonstrated that the largest burst of transcription occurs after a series of rapid and synchronous cell division cycles that partition the large maternal egg cytoplasm into thousands of smaller cells [4,5]. These experiments laid the groundwork for current investigations of how and to what degree ZGA timing is regulated by changes in the nuclear-to-cytoplasmic (N/C) ratio, developmental time, cell cycle duration, and cell size across diverse species [1]. A more recent area of great interest in the field is dissecting how changes in the 3D genome architecture during ZGA relate to changes in transcriptional output (reviewed in [6–8]). However, it is still unclear how all of these events work together to trigger ZGA at a precise developmental stage, and how these mechanisms differ across metazoans.

Here we summarize recent developments in the ZGA field in the context of two fundamental questions: 1) How is the genome primed for ZGA? and 2) What determines the timing of ZGA within and across species?

## Pioneer factors and histones prime the chromatin landscape for zygotic genome activation

### Pioneer factors create accessibility by peeling open the genome

**Figure 1:**
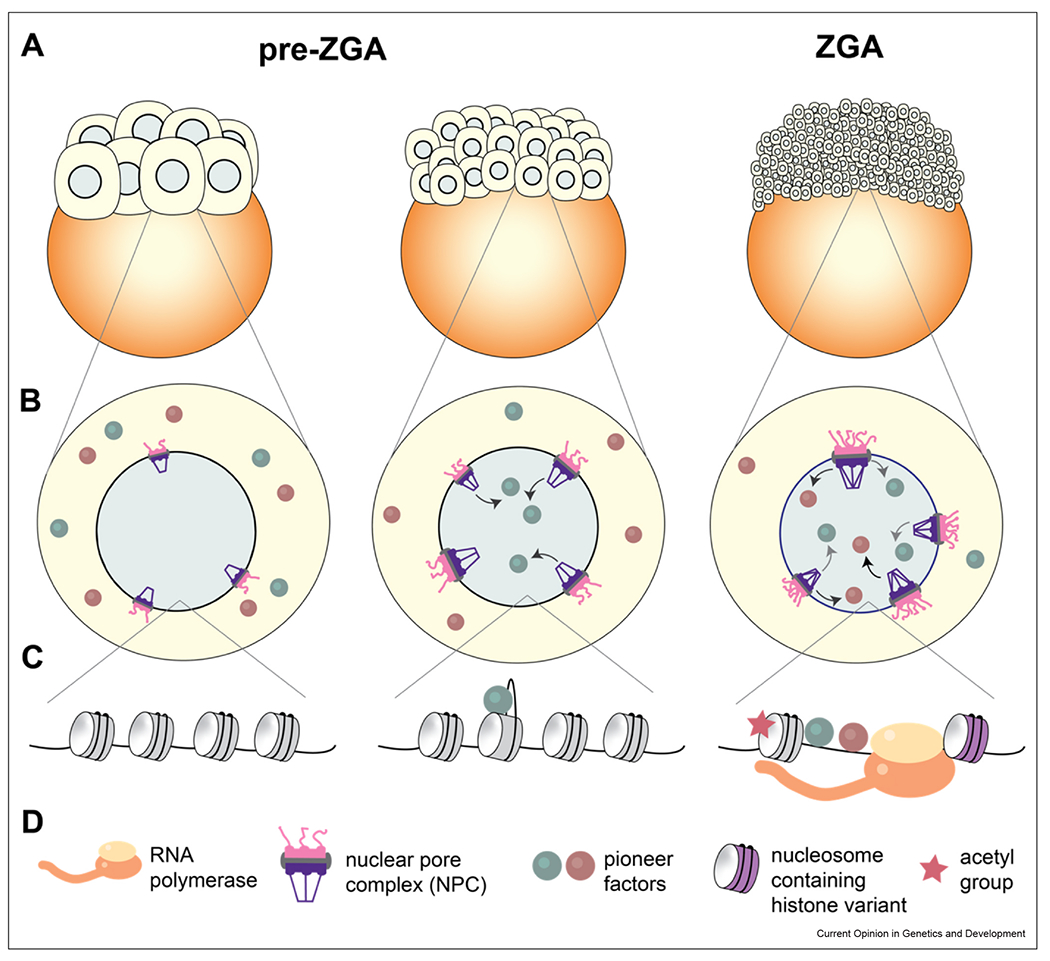
A series of chromatin remodeling events triggers ZGA. **(a)** ZGA occurs toward the end of a series of reductive cleavage divisions. **(b)** As cleavage divisions progress, NPCs increase in size and complexity [45••]. Due to differences in the affinity of pioneer factors for nuclear import machinery, the green pioneer factor is imported before the brown pioneer factor [46••]. **(c)** At the nucleosome level, pioneer factors peel open nucleosomal DNA at their target site, creating accessibility for downstream factors such as histone chaperones that exchange canonical histones with variants [32,33•], and histone acetyltransferases [18,30••]. Ultimately these events create an open genome structure that allows RNA Pol II to begin transcribing. **(d)** Legend for different molecules displayed.

Early models of ZGA proposed a role for maternally loaded transcriptional repressors that are progressively titrated away from the genome due to an increasing N/C ratio [4,5]. We now recognize that an additional barrier to activating a naïve genome is the ability of transcription factors to bind and create accessibility at promoters and enhancers (Figure 1c–d). There is emerging evidence that accessibility is initiated by pioneer factors, which bind to specific DNA sequences on nucleosomes and expose nearby regions of DNA that can then recruit additional activating factors such as chromatin remodeling enzymes [3,9]. The classic ZGA-specific pioneer factors include Pou5f3, Nanog and Sox19b in zebrafish [10,11]; Zelda in *Drosophila* [12]; and DUX [13–15], NFY [16], and more recently Nr5a2 [17••] in mouse. In all cases, depletion, inhibition, or knockout of one or more pioneer factors results in decreased transcription of zygotic genes accompanied by decreased chromatin accessibility at key regulatory regions.

Since the discovery of Zelda as a major regulator of ZGA in *Drosophila* 15 years ago [12], it has been a mystery how approximately half of Zelda-regulated zygotic genes remained accessible even when maternal Zelda was depleted. Also, the premature expression of Zelda was not sufficient to precociously activate the genome [18]. Recent studies have solved this conundrum by demonstrating bona fide pioneer activities for CLAMP [19•] and GAF (GAGA Factor) [20•], both of which bind and create accessibility at GA repeats near Zelda-regulated genes. The discovery of GAF as a pioneer factor is of particular interest as it was recently demonstrated that in *Drosophila* hemocytes, single molecules of GAF spend an unusually long time bound to chromatin (~140 s), 20–30-fold longer than other studied transcription factors [21••]. This ‘kinetic dominance’ allows GAF to occupy ~100% of its target sites at any given time [21••]. Unlike Zelda, GAF binds to developmental patterning genes during mitosis as well as in interphase [22], actively bookmarking these genes for expression through the cell cycle [23••]. Together these data suggest that the unique ability of GAF to persist on chromatin for long periods of time, and perhaps especially during mitosis, is important for establishing transcriptional memory during the many nuclear divisions of early embryogenesis.

The observation that multiple pioneer factors can co-exist at the same locus raises the question of if and how pioneer factors cooperate with one another to create accessibility across the genome. Two recent studies directly addressed this question using zebrafish by creating maternal knockouts of the three ZGA factors Nanog (*n*), Pou5f3 (*p*), and Sox19b (*s*) in various combinations [24••]. One group created a triple knockout mutant of all three factors (MZ*nps*) and asked if the loss of accessibility at zygotic genes could be rescued by injecting mRNAs encoding one or more of them. They found that while each factor alone had its own pioneering activity, Nanog was required to open more regions than the other two factors. At regions bound by all three factors, about a third required the presence of one factor for the others to bind. Another study comparing double (MZ*p*s) versus single knockout (MZ*s*) mutants suggested that Sox19b and Pou5f3 mostly act independently to open chromatin [25]. These results, similar to findings in *Drosophila* [19,20•], suggest that pioneer factors can act independently or cooperatively depending on the genomic context, with Nanog being the most crucial. Consistent with these observations, live imaging experiments in zebrafish showed that Nanog was the first of the three factors to co-localize with Pol II-enriched transcription hubs prior to ZGA, and embryos containing mutant Nanog no longer formed these hubs [26•]. In the future, it will be interesting to dissect the molecular mechanism behind this privileged role for Nanog during ZGA.

### The histone code is reshuffled during zygotic genome activation to establish an open chromatin landscape

As pioneer factors find their target sequences on nucleosomes, histones themselves are also being marked, exchanged and redistributed across the genome during early embryogenesis [27]. Activating histone marks and variants can either cooperate with pioneer factors to maintain open promoters and enhancers, or act independently to regulate ZGA (Figure 1). For example, the acetylation of histones loosens inter-nucleosomal contacts *in vitro* [28] and correlates with active transcription *in vivo* [29]. In mouse embryos, the inhibition of Brd4 and p300, the reader and writer for H3K27ac, respectively, resulted in aberrant ZGA, loss of chromatin accessibility, and developmental arrest [30••]. Injection of Brd4 and p300 into zebrafish embryos was sufficient to induce premature ZGA of some genes [31]. However, treatment of wild-type embryos with inhibitors of Brd4 and p300 was not sufficient to block chromatin opening at the sites that were most transcriptionally down-regulated in the MZ*nps* mutant [24••]. Together these results suggest that histone acetylation is necessary but not always sufficient to activate the genome and sometimes acts downstream of pioneer factor activity.

Histone variants also play a role in ZGA but potentially through a different pathway. In *Drosophila*, the histone variant H2A.Z was shown to mark 65% of zygotic genes before they are activated, and knockdown of the H2A.Z-specific chaperone Domino diminished ZGA [32••]. Importantly, genes regulated by H2A.Z were largely unoccupied by Zelda, and the knockdown of Domino did not affect Zelda binding, suggesting the H2A.Z pathway functions independently of Zelda to activate zygotic transcription [32••]. In mouse embryos, redistribution of histone variant H3.3 by the histone chaperone CAF1 is required for normal ZGA though underlying mechanisms are unclear [33•]. Examining the physical interplay between histones and pioneer factors at zygotic genes in the early embryo is an exciting area for future work.

## Events related to the cell cycle converge to trigger zygotic genome activation

Multiple mechanisms have been proposed to trigger ZGA including the titration of maternally loaded factors due to increasing N/C ratio [4,5,34–37], a threshold cell size [38] or nuclear size [39], developmental time [31] or cell cycle duration [40]. Here we summarize the most recent work characterizing the contributions of N/C ratio, cell cycle length and nuclear import on ZGA timing.

### Nuclear-to-cytoplasmic ratio and cell cycle duration independently contribute to zygotic genome activation

**Figure 2:**
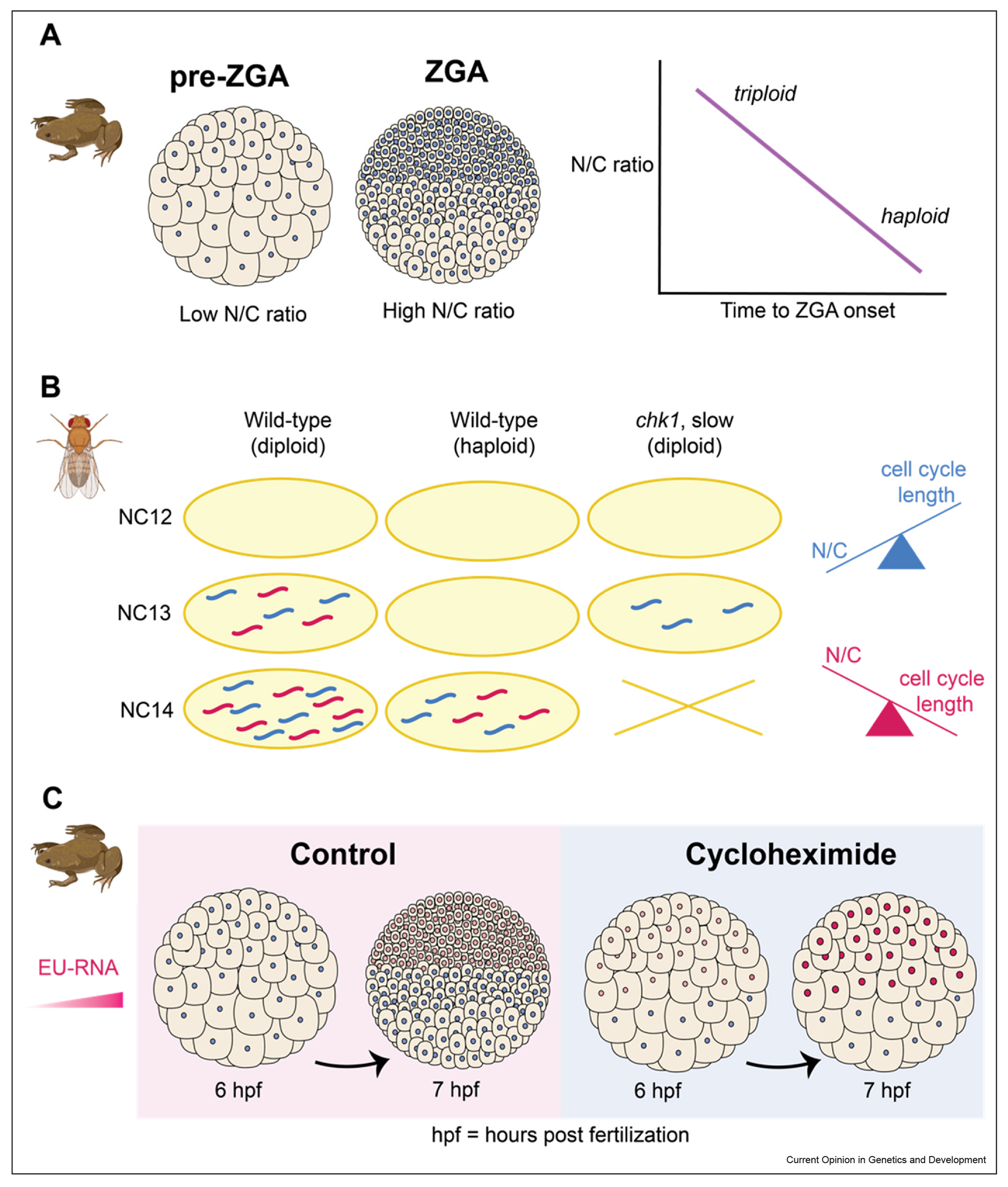
N/C ratio and cell cycle duration can independently trigger ZGA. **(a)** Left: As embryos progress through cleavage divisions, the increase in genome copies within a finite volume of maternal cytoplasm results in a dramatic increase in N/C ratio. Right: In *Xenopus*, decreasing genome content delays the onset of bulk ZGA [41•]. **(b)** In *Drosophila*, wild-type embryos initiate transcription at nuclear cycle 13 (NC13) while haploid embryos initiate transcription after an additional cell cycle, at nuclear cycle 14 (NC14). A diploid *chk1* mutant that has a slow NC13 phenocopies wild-type embryos for the activation of the blue transcript suggesting regulation by cell cycle length. However, the activation of the pink transcript is delayed, similar to the haploid embryos, suggesting regulation mainly by N/C ratio [43••]. **(c)** In *Xenopus*, the accumulation of total zygotic transcripts can be measured by the incorporation of fluorescent 5-ethynyluridine into nascent RNA. While control embryos initiate transcription once a threshold cell size is reached, treatment of pre-ZGA embryos with cycloheximide results in early onset ZGA in large cells [44••].

The exponential increase in genome copies within a finite volume of maternal cytoplasm results in a dramatic increase in N/C ratio as development proceeds (Figure 1a, Figure 2a), ultimately reaching a threshold N/C ratio that has been proposed to trigger ZGA [4,5]. Indeed, decreasing the total genome content three-fold using *Xenopus* cybrids (Figure 2a) [41] or two-fold using haploid *Drosophila* (Figure 2b) [43] delayed ZGA. However, the interpretation of this result is confounded by the long-standing observation that decreasing genome content causes embryos to undergo an additional cell division just before lengthening their cell cycle [4,42]. To distinguish how ZGA is influenced by N/C ratio versus cell cycle duration, one group used live imaging to track the accumulation of specific zygotic transcripts in *Drosophila* haploids, diploids, and a *chk1* mutant that shortens the nuclear cycle around the time of ZGA (Figure 2b) [43••]. For roughly half of the zygotic genes tested, the *chk1* mutant phenocopied the delay in ZGA observed in haploids, suggesting that the activation of these genes is regulated by cell cycle duration and not N/C ratio [43••], consistent with earlier work in *Drosophila* [40]. For another set of transcripts, the *chk1* mutant activated transcription at the same time as wild-type diploids, suggesting that N/C ratio was instead the major regulator of ZGA timing [43••]. In a separate study, prolonged treatment of *Xenopus* embryos with cycloheximide to induce interphase arrest triggered ZGA several hours earlier than in control embryos (Figure 2c) [44••], consistent with previous work in zebrafish [31]. Remarkably, the cycloheximide-treated embryos contained ~10-fold fewer cells than normal embryos, with a N/C ratio far lower than that of wild-type embryos during ZGA onset, yet still produced transcription profiles almost identical to untreated embryos [44••]. Taken together, these results demonstrate how N/C ratio and cell cycle duration can independently activate transcription. Future work will be required to identify underlying molecular mechanisms, which could be both gene-specific and species-specific.

### Nuclear import gates access of pioneer factors to the genome

An important missing piece of the puzzle is how ZGA timing is linked to chromatin remodeling activities that prime the genome for transcription. Two new studies introduce a potential role for nuclear import as a rate-limiting step for ZGA (Figure 1). One study in zebrafish used proteomics to quantify changes in nuclear proteins during the early cleavage stages [45••]. The authors discovered that among the proteins that increased most dramatically in nuclear abundance were key pioneer factors that trigger ZGA including Nanog, Sox19b and Pou5f3 [45••]. Using electron microscopy (EM) and immunofluorescence, they further showed that the nuclear pore complexes (NPCs) gradually increased in structural complexity leading up to ZGA [45••]. Together, these results suggest that the NPC assembly state can serve as a barrier to the entry of pioneer factors during early cleavage stages, thereby acting as a timer for ZGA. In a complementary story using similar approaches in *Xenopus*, the authors found that NPC proteins were among the first to be imported into the nucleus and that different transcription factors enter the nucleus at different times throughout the cleavage divisions [46••]. Using an extract-based biochemical assay, the authors went further to demonstrate that the timing of transcription factor entry correlates with their affinity for importin α/β, the major nuclear import machinery [46••]. This result is particularly exciting because it posits a simple biochemical model in which nuclear import gates the access of pioneer factors and other chromatin remodeling machinery to the genome, creating a temporally regulated sequence of events that ultimately trigger ZGA (Figure 1). Such a mechanism could also explain why increasing interphase duration can trigger ZGA as more time is allowed for importing key factors.

## Zygotic genome activation across evolution

ZGA timing differs dramatically across evolution. While flies, frogs and fish all initiate their major ZGA after 10–13 nuclear cycles [1,2], mice begin ZGA after only one cell division [47,48] and humans begin transcribing their genome at the one-cell stage [49•]. Despite these differences, we speculate that there are a few unifying principles for how ZGA is timed. First, since mammalian eggs are several orders of magnitude smaller than that of amphibian species that possess similar genome sizes, the N/C ratio at which ZGA initiates across species is not vastly different. We speculate that there is a universal N/C ratio threshold above which transcription will be active, and which could be tuned to different egg sizes among closely related species. For mammals with small eggs, this threshold is reached almost immediately upon fertilization, while for amphibians this threshold is only reached after multiple rounds of reductive cell divisions. There could also be a lower threshold for the N/C ratio below which transcription is silenced. Recent work comparing developmental timing across *Xenopus* species with a six-fold variation in genome size and 3.5-fold variation in egg size showed that early cleavage divisions and gastrulation occur with the same kinetics, suggesting that ZGA timing is conserved despite differences in the N/C ratio [50•]. A second unifying theme is that actively transcribing embryos, regardless of the species, have slow cell cycles on the timescale of hours or days, while rapidly cleaving embryos are transcriptionally silent [51]. One proposed explanation is that the machinery that silences or activates transcription, which is generally well-conserved across species, needs time to remodel its genomic targets [52–55]. In the future, it will be fascinating to test these ideas across a wide range of species, including close animal relatives [56•] to dissect the evolutionary history of ZGA.

## Summary and conclusions

The idea that a genome must be ‘awakened’ in an early embryo has puzzled researchers for more than 40 years [4,5]. Now with modern tools, we have a more detailed understanding of ZGA timing and mechanisms at the level of molecular players and specific genes. Armed with this knowledge, we envision that the next phase of ZGA research will integrate these findings into both universal and species-specific principles for transcriptional activation across molecular, cellular, and organismal scales.

## Data Availability

No data was used for the research described in the article.
